# Changes of COVID-19 Knowledge, Attitudes, Practices and Vaccination Willingness Among Residents in Jinan, China

**DOI:** 10.3389/fpubh.2022.917364

**Published:** 2022-05-20

**Authors:** Ning Jiang, Cheng Yang, Wenjing Yu, Liyan Luo, Xin Tan, Liping Yang

**Affiliations:** ^1^Department of Epidemiology, School of Public Health, Cheeloo College of Medicine, Shandong University, Jinan, China; ^2^Department of Project Office, Shandong Anke Blockchain Industrial Development Institute, Jinan, China; ^3^Leshan Community, Jinan, China

**Keywords:** COVID-19, vaccination willingness, vaccination hesitancy, KAP, change, China

## Abstract

**Background:**

Vaccine hesitancy is responsible for low vaccine coverage and increased risk of epidemics. The purpose of this study was to assess whether public knowledge, attitudes, practices, and willingness to vaccinate against COVID-19 have changed over time and at different stages of vaccination.

**Methods:**

Two consecutive surveys were conducted among residents of the Leshan Community in Jinan from May to June, 2021 (*n* = 423) (basic dose vaccination phase) and from December, 2021 to January, 2022 (*n* = 470) (booster vaccination phase). Randomly sampling was used in residents to complete an anonymous questionnaire. Chi-square test was used to compare the changes in knowledge, attitudes and practices of the subjects in different survey stages. Multivariable logistic regression analysis was used to explore factors related to vaccination hesitancy.

**Results:**

In the booster vaccination phase, protective behaviors (89.9%) of residents increased significantly compared with the basic vaccination phase (74.5%). Residents were more hesitant to receive booster doses than basal doses of COVID-19 vaccine (OR: 18.334, 95% CI: 9.021–37.262). Residents with other marital statuses (OR: 2.719, 95% CI: 1.632–4.528), negative attitudes toward government measures were more hesitant to get vaccinated (OR: 2.576, 95% CI: 1.612–4.118). People who thought their physical condition was very good or good were more likely to be vaccinated than those who thought they were in fair or poor health (OR: 0.516, 95% CI: 0.288–0.925; OR: 0.513, 95% CI: 0.295–0.893). Young people inclined to use new media (such as WeChat and microblog) to obtain information, while the elderly inclined to use traditional methods (such as television). Government propaganda, residents' perception of the importance of vaccines and the risk of disease were the main reasons for accelerating residents to vaccinate. The main reasons affecting residents' lack of vaccination were contraindications to the vaccine or inconvenient time for vaccination.

**Conclusions:**

Vaccine hesitancy increased significantly with change in vaccination stage. Strategies should be adopted to increase vaccination coverage such as improving the convenience of vaccination, promoting through multiple channels.

## Introduction

Coronavirus disease 2019 (COVID-19) broke out in December 2019, in Wuhan, Hubei and quickly spread across China, becoming a major global public health problem. The World Health Organization declared COVID-19 a public health emergency of international concern on January 30, 2020 (1). The COVID-19 epidemic has lasted more than 2 years. As of 13 March 2022, over 455 million confirmed cases and over 6 million deaths have been reported globally (2). COVID-19 seriously threatens people's physical and mental health, affects the social order, and hinders countries' economic development (3–5).

Fortunately, the successful development of specific medicine provides help for the treatment of COVID-19, but the role of vaccines in preventing the epidemic of infectious diseases is irreplaceable. vaccines have played critical roles in human struggles against major infectious diseases such as smallpox, polio, rabies, typhoid, plague and many more (6). As of April 2022, there are 68 vaccines in Phase 3 trials globally, 36 of which have been approved for use in at least one country (7). Only when a high rate of vaccination is achieved can an immune barrier be built (8). However, many previous studies have demonstrated vaccine hesitancy in the population (9, 10). And the acceptance of vaccines also varies between countries (11). Vaccine hesitancy refers to delay in acceptance or refusal of vaccination despite availability of vaccination services (12). Vaccine hesitancy is believed to be responsible for decreasing vaccine coverage and an increasing risk of vaccine-preventable disease outbreaks and epidemics (13).

A previous Chinese study investigated guardians' willingness to get COVID-19 vaccine for their children aged 3–17 (14). But parents may have different attitudes about vaccinations for themselves and their children. We surveyed the attitudes of adults toward vaccinating themselves.

The purpose of our study was to assess whether and how the public's knowledge, attitudes, practices, and willingness to vaccinate against COVID-19 changed by time and different stages of vaccination, and to analyse the influencing factors associated with vaccination hesitancy. By focusing on the weaker aspects of residents' knowledge, negative attitudes and unhealthy daily practices, targeted advertising and education can be adopted to increase the comprehensive understanding of the emerging infectious disease, eliminate panic and improve awareness of prevention, which are very important to the stability of social order.

Jinan is located in Eastern China, connecting to the Beijing-Tianjin-Hebei urban agglomeration in the north and the Yangtze River Delta economic circle in the south. It is a national historical and cultural city. The total population of Jinan City is 9.2 million, of which 7.42 million are 18 years old and above. The Leshan Community has complex socio-demographic characteristics and locates in the center of Jinan. It is a representative community that can be regarded as a miniature Jinan.

## Materials and Methods

### Study Design

We conducted two surveys on residents of the Leshan Community in Jinan City at different stages of COVID-19 vaccination. The first survey was conducted from 24 May to 12 June 2021 (first dose vaccination phase). The second survey was conducted from 30 December, 2021 to 9 January, 2022 (booster vaccination phase). Random sampling of residents was used to complete an anonymous questionnaire. $n=\frac{z_{\left(1-\alpha \right)/2}^{2}pq}{d^{2}}\times deff$ was used to calculate the sample size. The vaccination rate at the time of the first survey was about 70% in Jinan, so *p* = 0.7, *q* = 0.3, *d* = 0.1p, *deff* = 2, α = 0.05. Therefore, the sample size was 330. Five hundred people were randomly selected in the research. First, 10 of the 59 residential buildings were randomly selected, and then 50 persons lived in the selected residential buildings were randomly selected. In the first survey, 423 people responded effectively, with an effective response rate of 84.6%. In the second survey, 470 people responded effectively, with an effective response rate of 94.0%.

### Inclusion and Exclusion Criteria

Residents aged 18 or older who understood the content of the study, had no barriers to communication or understanding, and agreed to participate in the study.

### Questionnaire Content

The content of the anonymous questionnaire was designed with reference to the prevention and control knowledge of the National Health Commission website and the “New Coronavirus Pneumonia Diagnosis and Treatment Protocol (Trial Version 8)”(15, 16). The questionnaire content included five main features. The first referred to the socio-demographic characteristics of the subject (gender, marital status, age group, occupation, and education level). The second involved respondents' knowledge regarding COVID-19, including the pathogen and epidemiology, clinical manifestations of the disease, daily protection and prevention (one point was awarded for correct answers, no points for incorrect answers, the total score of knowledge toward COVID-19 is 10. The total score less than the mean value was interpreted as poor knowledge, and the total score greater than or equal to the mean value was interpreted as good knowledge). The third was the section on attitudes regarding government's prevention and control measures that adopted use of the 5-point Likert scale (a total of 12 points, the total score less than the mean value was interpreted as negative attitudes, and the total score greater than or equal to the mean value was interpreted as positive attitudes.) The fourth was the section investigating the public's daily protective practices which contains eight items, the total score less than the mean value was interpreted as poor practice, and the total score greater than or equal to the mean value was interpreted as good practice. Finally, the section on the COVID-19 vaccine investigated COVID-19 vaccination willingness and reasons. Residents were asked if they would be willing to be vaccinated against COVID-19, and if they answered unwilling or unsure, they were considered vaccine hesitant. Vaccine hesitancy is not considered to exist if the answer is yes. We then asked vaccine hesitant people why they were reluctant to get vaccinated, and asked people willing to get vaccinated what motivated them.

### Quality Control

The survey questionnaire in electronic form was sent to residents by community staff. To ensure integrity of the data, the electronic questionnaire could only be submitted after all questions had been answered. WeChat was used to verify the identity of the respondents and as a way of logging in to answer the questionnaire. Each WeChat account could only be submitted once to avoid repeated answers. Questionnaires that took <180 s were judged to be invalid. Considering the infrequent use of mobile phones by the elderly, we conducted a face-to-face interview with them. Questionnaires were administered and filled out by investigators who had received uniform training to ensure the quality. Before the formal survey, we conducted a preliminary survey of 50 residents to assess the validity and understandability of the questionnaire. Then, some adjustments were made based on the pilot study. Likert5 scale was adopted in the attitude part, so the Cronbach's alpha of the attitude was 0.857. For the parts of knowledge and practice, pre-investigation and expert evaluation were both used to ensure the quality of the questionnaire.

### Statistical Analysis

Statistical analysis was performed using SPSS 26.0 software. The composition ratio [n (%)] was used to describe general demographic characteristics and vaccination status. Chi-square test was used to compare the changes of sampling subjects' knowledge, attitude and practice (KAP) and willingness to vaccinate against COVID-19 in different survey stages. Logistic regression analysis was used to explore factors related to vaccination hesitancy. Independent predictors of vaccination hesitancy were assessed using binary logistic regression models. Then, the variables with *p* < 0.2 in the univariate logistic regression were included in the multivariable logistic regression model, and the model was constructed by the likelihood ratio test method. The model fitting effect was assessed using the Hosmer-Lemeshow goodness of fit test. The statistical significance level was set at *p* < 0.05.

### Ethics

The research protocol was approved by the Public Health Ethics Committee of Shandong University (LL20211201). Our research has been carried out in accordance with the principles stipulated by Helsinki.

## Results

### Socio-Demographic Characteristics

Table 1 shows the characteristics of the respondents in two surveys. There were no significant differences among participants in terms of gender, age, marital status, occupation, educational level, chronic disease and physical conditions. There were 423 and 470 respondents in the first and second surveys, respectively. According to the level of infection risk and the nature of work, we classified occupations into the following groups: “high risk of infection” (customs officer, medical staff, transportation staff), “occupation in key positions” (teacher, public service industry, government employees), and “other” (students, retirees, enterprise employees). Among survey respondents, more than half were women. The majority of participants were married (84.7–87.2%). Overall, 57.2–58.8% residents had a college and undergraduate degrees or above. Among occupations, “other” accounted for the largest proportion (78.5–82.5%) (Table 1).

**Table 1:** Comparison of resident characteristics in two surveys.

		**Basic vaccination phase (*n* = 423)**	**Booster vaccination phase (*n* = 470)**	
**Variables**	**Category**	***n*** **(%)**	***n*** **(%)**	***P***
Sex				0.554
	Male	172(40.7)	182 (38.7)	
	Female	251(59.3)	288 (61.3)	
Age				0.731
	18–30	30 (7.1)	33 (7.0)	
	31–40	114 (27.0)	113 (24.0)	
	41–50	96 (22.7)	108 (23.0)	
	51–60	58 (13.7)	59 (12.6)	
	>60	125 (29.6)	157 (33.4)	
Marital status				0.274
	Married	369(87.2)	398 (84.7)	
	Others	54(12.8)	72 (15.3)	
Education status				0.537
	Middle school and below	69 (16.3)	94 (20.0)	
	High school and technical secondary school	105 (24.8)	107 (22.8)	
	College and Undergraduate	212 (50.1)	228 (48.5)	
	Master and above	37 (8.7)	41 (8.7)	
Occupation				0.257
	High risk of infection	13 (3.1)	22 (4.7)	
	Key occupations	61 (14.4)	79 (16.8)	
	Others	349 (82.5)	369 (78.5)	
Chronic disease				0.549
	Yes	132 (31.2)	138 (29.4)	
	No	291 (68.8)	332 (70.6)	
Physical conditions				0.654
	Very good	139 (32.9)	168 (35.7)	
	Good	217 (51.3)	229 (48.7)	
	General and low	67 (15.8)	73 (15.5)	
Knowledge				0.146
	Good	306 (72.3)	319 (67.9)	
	Poor	117 (27.7)	151 (32.1)	
Attitude				0.141
	Positive	324 (76.6)	414 (80.6)	
	Negative	99 (23.4)	91 (19.4)	
Practice				<0.001
	Good	315 (74.5)	422 (89.9)	
	Poor	108 (25.5)	48 (10.2)	
Vaccine willingness				<0.001
	Willingness	414 (97.9)	345 (74.5)	
	Hesitancy	9 (2.1)	120 (25.5)	

*Chi-square test, P < 0.05*.

### Ways to Obtain Information About COVID-19

**Figure 1:**
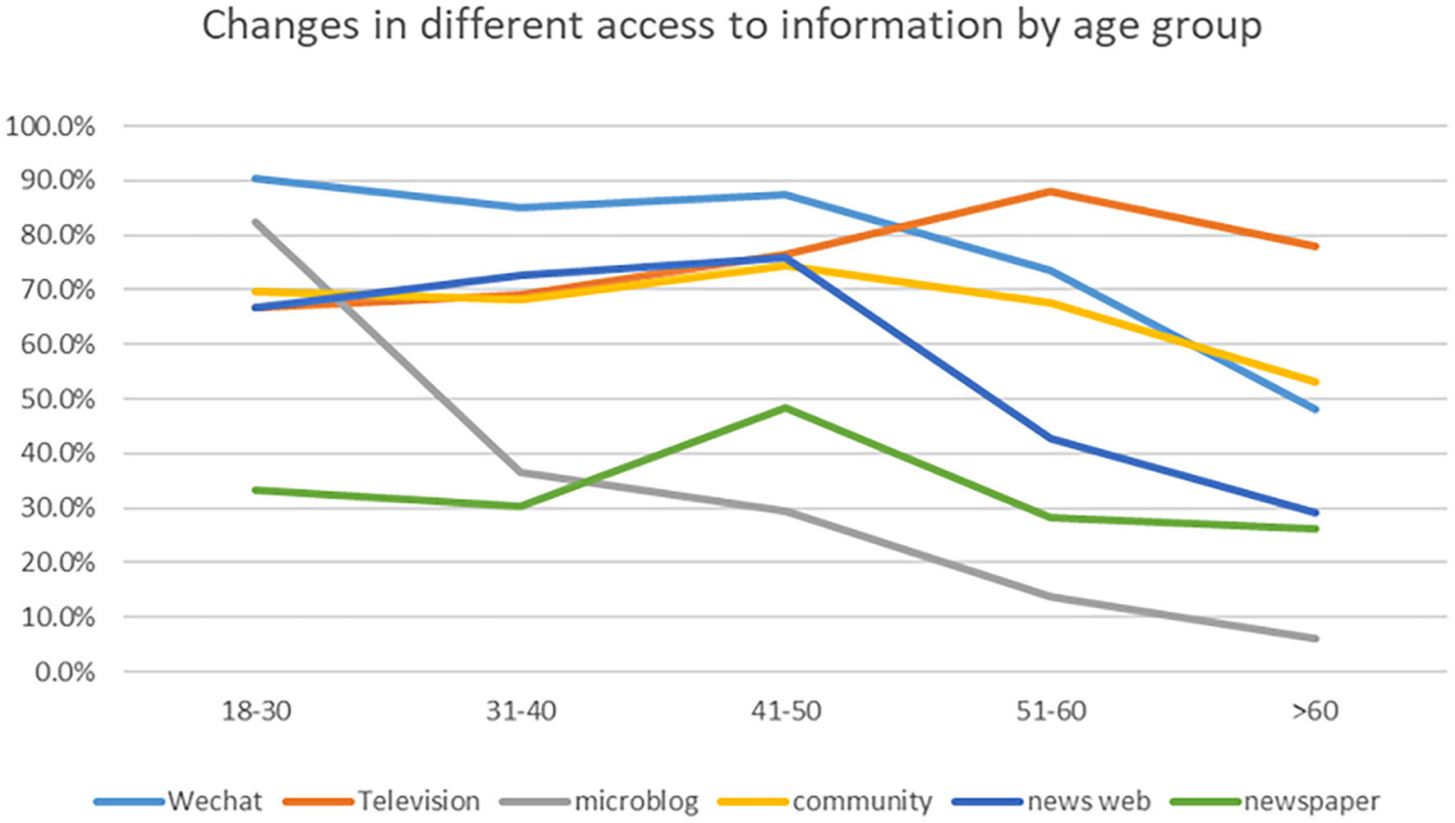
Changes in different access to information by age group.

Access to information is age-related. Furthermore, the age composition of the two surveys was similar. Therefore, data from the two surveys were combined to reflect an overall picture of ways to obtain information about COVID-19. Television (75.9%), WeChat (72.8%), community advertising (64.9%), and news websites (55.3%) were identified as the main ways for residents to obtain information. People aged 18–30 most often used WeChat (90.5%) and microblog (82.5%). People aged 31–40 and 41–50 years old used WeChat most frequently, 85.0% and 87.3%, respectively. People aged 51–60 and over the age of 60 used television most often, at 88.0% and 78.0%, respectively. With the increase of age, the number of residents who obtain information through WeChat and microblog gradually decreases. Residents who access information through Television gradually increase with age (Figure 1).

### Knowledge Regarding COVID-19

In the two surveys, 72.3% and 67.9% residents had good knowledge of COVID-19, respectively. We list the correct rate of residents' knowledge about the COVID-19 in the two surveys (Table 2). The correct answer rates of the questions on the COVID-19 knowledge questionnaire were 65.2–97.4%, 60.0–96.8%, respectively. In the stage of booster vaccination, the proportion of respondents who believed that patients with COVID-19 may have nasal congestion, runny nose, sore throat rose to 86.8%, compared with 70.2% in the basic vaccination phase (*p* < 0.001). The proportion of respondents who believed that critical illnesses are more common in the elderly, and in those with underlying diseases rose to 86.4%, compared with 74.2% in the basic vaccination phase (*p* < 0.001). However, the correct perception that wearing multiple masks and antibiotics did not prevent COVID-19 decreased from 78.5% to 60.0% and from 72.1% to 40.8%, respectively (*p* < 0.001).

**Table 2:** Comparison of correct knowledge about COVID-19 between two surveys.

**Questions**	**Basic vaccination phase** ***n* (%)**	**Booster vaccination phase** ***n* (%)**	***P***
COVID-19 is mainly transmitted through the respiratory tract (yes)	412 (97.4)	454 (96.6)	0.484
Asymptomatic infection is contagious (yes)	393 (92.9)	432 (91.9)	0.576
COVID-19 mainly invaded the lungs (yes)	391 (92.4)	415 (88.3)	0.037
Alcohol concentration to eliminate the new coronavirus (75%)	328 (77.5)	341 (72.6)	0.086
Fever, dry cough, and fatigue are the main manifestations of COVID-19 (yes)	402 (95)	455 (96.8)	0.179
Patients with COVID-19 may have nasal congestion, runny nose, sore throat and other symptoms (yes)	297 (70.2)	408 (86.8)	<0.001
Critical illnesses are more common in the elderly, and in those with underlying diseases (yes)	314 (74.2)	406 (86.4)	<0.001
Multiple masks have better protection effect (no)	332 (78.5)	282 (60.0)	<0.001
Antibiotics can prevent COVID-19 (no)	305 (72.1)	234 (49.8)	<0.001
There have specific drugs for the treatment of COVID-19 (no)	276 (65.2)	310 (66.0)	0.824

*Chi-square test, P < 0.05*.

### Attitudes About Government Measures During Lockdown Period

In the two surveys, 76.6% and 80.6% residents displayed a positive attitude about government measures, respectively. Compared with the basic vaccination phase, the booster vaccination phase found that residents were found to be more willing to “very agree” with wearing masks in public places (87.9% vs. 78.7%), taking their temperature when entering supermarkets (85.5% vs. 72.6%), and self-isolating at home during the lockdown period (84.3% vs. 75.2%) (Table 3).

**Table 3:** Comparison of attitudes toward government measures between two surveys.

**Attitudes**	**Basic vaccination phase** **(*N* = 423)**	**Booster vaccination phase** **(*N* = 470)**	***P***
**Wearing masks in public places**			<0.001
Disagree/general	4 (0.9)	6 (1.3)	
Agree	86 (20.3)	51 (10.9)	
Very agree	333 (78.7)	413 (87.9)	
**Self-isolating at home during the lockdown period**			<0.001
Disagree/general	3 (0.7)	16 (3.4)	
Agree	102 (24.1)	58 (12.3)	
Very agree	318 (75.2)	396 (84.3)	
**Taking their temperature when entering supermarkets**			<0.001
Disagree/general	9 (2.1)	12 (2.6)	
Agree	107 (25.3)	56 (11.9)	
Very agree	307 (72.6)	402 (85.5)	

*Chi-square test, P < 0.05*.

### Protective Practices Toward COVID-19

In the two surveys, 74.5% and 89.9% of the residents maintained “good” protective measures, respectively. Compared with the basic vaccination phase, the booster vaccination phase found that residents were more frequently washing their hands in daily life (99.1% vs. 97.2%), maintaining social distancing (94.9% vs. 87.0%), and cleaning their houses (97.7% vs. 91.3%). In the basic vaccination phase, majority of respondents (87.0%) maintained more than one meter of distance when communicating with others, and 91.3% cleaned their home every day. The implementation rates for six other behaviors were all >95.0%. In the booster vaccination phase, the implementation rate of residents' behavior increased and the gap narrowed. The implementation rates for all behaviors were all ≥94.9% (Table 4).

**Table 4:** Comparison of protective behaviors toward government measures between two surveys.

**Practice**	**Basic vaccination phase (*n* = 423)**	**Booster vaccination phase (*n* = 470)**	***P***
**Wearing mask in public place**			0.315
Yes	403 (95.3)	454 (96.6)	
No	20 (4.7)	16 (3.4)	
**Washing hands in daily life**			<0.026
Yes	411 (97.2)	466 (99.1)	
No	12 (2.8)	4 (0.9)	
**Covering mouth and nose when coughing or sneezing**			0.227
Yes	419 (99.1)	461 (98.1)	
No	4 (0.9)	9 (1.9)	
**Opening windows every day for ventilation**			0.100
Yes	423 (100.0)	467 (99.4)	
No	0 (0.0)	3 (0.6)	
**Social distance**			<0.001
Yes	368 (87.0)	446 (94.9)	
No	55 (13.0)	24 (5.1)	
**Reducing the number of gatherings**			1.000
Yes	414 (97.9)	460 (97.9)	
No	9 (2.1)	10 (2.1)	
**Cleaning home every day**			<0.001
Yes	386 (91.3)	459 (97.7)	
No	37 (8.7)	11 (2.3)	
**Eating a balanced diet**			0.189
Yes	409 (96.7)	461 (98.1)	
No	14 (3.3)	9 (1.9)	

*Chi-square test, P < 0.05*.

### COVID-19 Vaccination Willingness and Situation

In the first survey, 414 (97.9%) residents intended to receive the COVID-19 vaccine, 310 (74.9%) of which had received the first dose of the vaccine, and 110 cases (26.6%) were fully vaccinated. Among those vaccinated, 248 (80.0%) received inactivated vaccines, 93 (37.5%) of which were fully vaccinated, and 155 (62.5%) only received the first dose. In the second survey, 350 (74.5%) residents would like to receive a booster vaccine. 25.5% of residents were skeptical about booster vaccine. Among the 443 residents who received the COVID-19 vaccine, 222(50.1%) residents had received booster dose of COVID-19 vaccine.

### Factors Associated With COVID-19 Vaccine Hesitancy

Logistic regression was performed between the vaccine demand group and vaccine delay group to identify the influencing factors of vaccination hesitancy.

In univariate logistic regression analysis, we found that marital status, physical conditions, number of surveys and attitudes were statistically significantly correlated to vaccine hesitancy. In the multivariable logistic regression analysis (Table 5), residents' willingness to receive a booster vaccine showed higher hesitancy than their willingness to receive the basic dose (OR: 18.334, 95% CI: 9.021–37.262). Residents with other marital statuses were more hesitant to get vaccinated than married people (OR: 2.719, 95% CI: 1.632–4.528). People with negative attitudes toward government measures were more hesitant to get vaccinated (OR: 2.576, 95% CI: 1.612–4.118). People who thought their physical condition was very good or good were more likely to be vaccinated than those who thought they were in fair or poor health (OR: 0.516, 95% CI: 0.288–0.925; OR: 0.513, 95% CI: 0.295–0.893).

**Table 5:** Logistic regression analysis of factors affecting COVID-19 vaccine hesitancy of survey subjects.

**Variables**	**Univariate OR (95%CI)**	***P***	**Multivariable OR (95%CI)**	***P***
**Age group**		0.074		
>60	Reference			
18–30	1.268 (0.639–2.514)			
31–40	0.859 (0.532–1.386)			
41–50	0.472 (0.265–0.838)			
51–60	0.772 (0.419–1.424)			
**Gender**		0.979		
Male	Reference			
Female	1.005 (0.686–1.472)			
**Marital satus**		<0.001		<0.001
Married	Reference		Reference	
Other	2.899 (1.861–4.515)		2.719 (1.632–4.528)	
**Occupation**		0.089		
Others	Reference			
High risk of infection	0.502 (0.151–1.667)			
Key occupations	0.548 (0.299–1.004)			
**Education**		0.138		
Middle school and below	Reference			
High school and technical secondary school	1.546 (0.735–3.251)			
College and Undergraduate	0.965 (0.457–2.040)			
Master and above	0.888 (0.443–1.783)			
**Physical conditions**		0.019		0.042
General and low	Reference		Reference	
Good	0.505 (0.310–0.822)		0.513 (0.295–0.893)	0.018
Very good	0.557 (0.333–0.933)		0.516 (0.288–0.925)	0.026
**Chronic disease**		0.098		
No	Reference			
Yes	1.391 (0.940–2.056)			
**Vaccination phase**		<0.001		<0.001
Basic vaccination phase	Reference		Reference	
Booster vaccination phase	15.771 (7.893–31.512)		18.334 (9.021–37.262)	
**Knowledge**		0.086		
Good	Reference			
Poor	1.408 (0.952–2.083)			
**Attitude**		<0.001		<0.001
Positive	Reference		Reference	
Negative	2.191 (1.460–3.289)		2.576 (1.612–4.118)	
**Practice**		0.893		
Good	Reference			
Poor	0967(0.589–1.586)			

*Logistic regression analysis, P < 0.05*.

### Reasons Affecting COVID-19 Vaccination

In the second survey, we investigated what motivated residents to receive booster vaccine in the vaccinated group and the refusal reasons for vaccine hesitancy in the hesitant group. Among 350 people who would like to be vaccinated, most people believed that the reasons for promoting vaccination were: “the government's propaganda,” “to protect family/friends/colleagues from infection,” that “job requirements” and “concern about contracting COVID-19” accounted for 66.6%, 60.9%, 56.9%, and 56.6%, respectively. Among 120 vaccine hesitant people, the top two reasons were “inconvenient time for vaccination” and “there are contraindications for vaccination”, which accounted for 22.5% and 17.5% respectively.

## Discussion

To investigate changes in public knowledge, attitudes, practices, and willingness to vaccinate against COVID-19 in Jinan, two consecutive surveys were conducted during the basic vaccination phase (May–June 2021) and the booster vaccination phase (December–January 2021). Our research showed that, on the whole, knowledge and attitudes of residents about COVID-19 did not change much between the two phases, but behaviors were more positive in the booster vaccination phase than in the basic vaccination phase. Residents were more hesitant to get booster dose than the basic dose. Marital status, physical conditions, investigation stage, and attitudes were the influencing factors of vaccine hesitancy.

One research was conducted online during the first wave and third wave of the local epidemic in 2020 in Hong Kong, China. The results showed that with the time changes, the vaccination willingness declined but the compliance with personal protective behaviors increased (17). It is consistent with our research results.

In the basic vaccination phase, it was found that the research subjects had good knowledge of the epidemiological characteristics and main clinical symptoms of COVID-19, but knowledge about other special clinical symptoms and protective measures of COVID-19 was bad. The similar situations were also appeared in the booster vaccination phase and other studies (18, 19). Obviously, residents have not systematically mastered the relevant knowledge regarding COVID-19, resulting in knowledge weaknesses and blind spots. Therefore, it is necessary to strengthen the depth of residents' health education by formulating a systematic and comprehensive learning plan, thus increasing the awareness rate of COVID-19 knowledge.

After the outbreak of COVID-19, the government adopted many measures which were accepted by the vast majority of residents. This study showed that the attitudes of residents in the booster vaccination phase were similar to those in the basic vaccination phase, and most of them (76.6–80.6%) maintained positive attitudes. During the pandemic of COVID-19, Chinese government has been taking many strategies and measures to prevent, control and therapy the emerging infectious disease. As a result, majority of residents have benefits from the measures, and they believe China can do well against the virus, so they can have positive attitudes against COVID-19.

In the two surveys, more than 95% of respondents wore masks in public places where people gather. Using masks can protect healthy people from infection and reduce the spread of the virus (20, 21). However, a survey in Malaysia showed that 51.2% of residents wear masks when went go out. They might believe only people who have symptoms of COVID-19 or similar diseases need to wear medical masks (22). Regarding self-care, more than 96% of respondents in two surveys reflected they performed strengthen exercise, rested regularly. An online survey revealed that the response rate of participating in physical exercise was relatively low (61.7%) during the quarantine period (23). Maybe due to different periods of investigation, we conducted the research during the normalization of the epidemic. While in the quarantine period, staying at room might lead to less physical exercise. According to a survey in Saudi Arabia, 98% of the public adopted social distancing, similar to the results of the booster vaccination phase of this study (24). In addition, our research found residents had better protective behaviors in the booster vaccination phase than the basic vaccination phase (74.5% vs. 89.9%, *p* < 0.001). May be due to the government's emphasis on the importance of protective behavior in preventing COVID-19. With the pandemic of COVID-19, residents' awareness of protective was increasing.

This study showed COVID-19 vaccination willingness among community residents was 97.6% during the basic vaccination phase. It is higher than the willingness (91.7%, 91.9%, 88.6%) of Chinese residents to be vaccinated in the survey from March to June, November– December 2020 (11, 25, 26). In the stage of booster vaccination, it was 74.5% of the COVID-19 vaccination willingness among residents. It was similar to the willingness of Chinese residents to be vaccinated (75.2%) surveyed in April–May 2021 (27). In the basic vaccination phase, 2.1% of residents were hesitant to vaccinate, and the proportion of hesitant to vaccinate increased to 25.5% in the stage of booster vaccination. It was more difficult to vaccinate eligible residents in China with the booster dose than the basic dose (*p* < 0.001). The willingness of residents to receive the booster vaccine was lower than the willingness to receive the basic vaccine.

This study found that people with other marital statuses were more hesitant to get vaccinated than married people which was consistent with other researches (28, 29). Married residents paid more attention to the safety of their mate, children and other family members. They were vaccinated in order to protect the safety of themselves and their families. It indicated that family responsibility drove vaccination.

Additionally, our research showed that respondents with negative attitudes toward government protective measures were more hesitant to get vaccinated than those with general attitudes, which reflected the transformation of attitudes into behaviors. People who thought he or she was healthy have a higher vaccination rate than those with ordinary or poor health, similar to previous survey results (30). Vaccine hesitancy of COVID-19 is complex, varying across time, place.

This research showed that government calls and perceptions of disease risk promoted vaccination. The perceived importance of vaccines, the risk perception of the disease, and the accessibility and convenience of vaccination services are all important factors affecting vaccination (13). With a higher degree of trust in government information, residents were more likely to receive vaccine against COVID-19.

The study showed that young people were more inclined to use new media (such as WeChat and microblog) to obtain information, while the elderly were more inclined to use traditional methods (such as television). Different age had different levels of access to information, consistent with a study in Malaysia (31). Using traditional methods to obtain information could increase the possibility of vaccination, most likely because they insist on high-quality information sources and share fact-based information (32). People could get information quickly and easily on news media, but it might also be a source of misinformation (33). Therefore, government departments should continue to use traditional media channels, and try to promote high-quality information to new media platforms to increase the vaccination acquisition rate.

Results indicated 21.7% had registered to receive the vaccination but had not yet been notified to do so. So reasonable and standardized vaccination services would be able to promote vaccination. Previous surveys showed that the main reason for hesitation in vaccines was concern about safety and effectiveness (30, 34, 35). In this study, only a small percentage (5.8%) of those who did not receive vaccination stated because they doubted the effectiveness and safety of the vaccine, it indicated that after a period of advertising and education by the government and related agencies, most of the public were no longer concerned about the safety and effectiveness of the vaccine.

We used longitudinal research to investigate public changes on knowledge, attitudes, practices, and willingness to vaccinate against COVID-19 in Jinan, China. Considering the infrequent use of the Internet by older adults, a combination of online and face-to-face surveys were used to make the sample more representative. But the research was conducted in one region, so the conclusions may not be generalizable to other regions.

In conclusion, different propaganda channels can be adopted for differing groups of residents. Education should be focused particularly on those residents who have inadequate knowledge about COVID-19 to increase the comprehensive understanding of the emerging infectious disease. More measures should be adopted to increase vaccination coverage, such as expanding the number of alternative vaccines, improving vaccine efficiency, researching vaccines to deal with mutant strains. Eliminating the spread of COVID-19 requires not only vaccination, but also maintaining good practices.

## Data Availability Statement

The raw data supporting the conclusions of this article will be made available by the authors, without undue reservation.

## Ethics Statement

The studies involving human participants were reviewed and approved by Public Health Ethics Committee of Shandong University. Written informed consent for participation was not required for this study in accordance with the national legislation and the institutional requirements.

## Author Contributions

NJ and LY designed research. NJ, CY, WY, LL, and XT curated data collection. NJ analyzed data and wrote the paper. All authors contributed to the article and approved the submitted version.

## Conflict of Interest

The authors declare that the research was conducted in the absence of any commercial or financial relationships that could be construed as a potential conflict of interest.

## Publisher's Note

All claims expressed in this article are solely those of the authors and do not necessarily represent those of their affiliated organizations, or those of the publisher, the editors and the reviewers. Any product that may be evaluated in this article, or claim that may be made by its manufacturer, is not guaranteed or endorsed by the publisher.

## References

[1] Organization WH. Statement on the second meeting of the International Health Regulations (2005) Emergency Committee regarding the outbreak of novel coronavirus (2019-nCoV). Available online at: https://bit.ly/2zc56Vk

[2] Organization WH. COVID-19 Weekly Epidemiological Update. Available online at: https://www.who.int/publications/m/item/weekly-epidemiological-update-on-covid-19-15-march-2022

[3] Nicola M Alsafi Z Sohrabi C Kerwan A Al-Jabir A Iosifidis C. The socio-economic implications of the coronavirus pandemic (COVID-19): a review. Int J Surg. (2020) 78:185–93. 10.1016/j.ijsu.2020.04.018 32305533 PMC7162753

[4] Li Q Miao Y Zeng X Tarimo CS Wu C Wu J. Prevalence and factors for anxiety during the coronavirus disease 2019 (COVID-19) epidemic among the teachers in China. J Affect Disord. (2020) 277:153–8. 10.1016/j.jad.2020.08.017 32828002 PMC7425543

[5] Flor LS Friedman J Spencer CN Cagney J Arrieta A Herbert ME. Quantifying the effects of the COVID-19 pandemic on gender equality on health, social, and economic indicators: a comprehensive review of data from March, 2020, to September, 2021. Lancet. (2022). 10.1016/S0140-6736(22)00008-3 35247311 PMC8890763

[6] Harrison EA Wu JW. Vaccine confidence in the time of COVID-19. Eur J Epidemiol. (2020) 35:325–30. 10.1007/s10654-020-00634-3 32318915 PMC7174145

[7] Team MCVT. Vaccines candidates in clinical trials. Available online at: https://covid19.trackvaccines.org/vaccines/#approved

[8] Fine P Eames K Heymann DL. “Herd immunity”: a rough guide. Clin Infect Dis. (2011) 52:911–6. 10.1093/cid/cir007 21427399

[9] Freeman D Loe BS Chadwick A Vaccari C Waite F Rosebrock L. COVID-19 vaccine hesitancy in the UK: the Oxford coronavirus explanations, attitudes, and narratives survey (Oceans) II. Psychol Med. (2020):1–15. 10.1017/S0033291720005188 33305716 PMC7804077

[10] Murphy J Vallieres F Bentall RP Shevlin M McBride O Hartman TK. Psychological characteristics associated with COVID-19 vaccine hesitancy and resistance in Ireland and the United Kingdom. Nat Commun. (2021) 12:29. 10.1038/s41467-020-20226-9 33397962 PMC7782692

[11] Lazarus JV Ratzan SC Palayew A Gostin LO Larson HJ Rabin K. A global survey of potential acceptance of a COVID-19 vaccine. Nat Med. (2021) 27:225–8. 10.1038/s41591-020-1124-9 33082575 PMC7573523

[12] MacDonald NE. Hesitancy SWGoV. Vaccine hesitancy: definition, scope and determinants. Vaccine. (2015) 33:4161–161:5e and 1016/j.vaccine.2015.04.036 25896383 10.1016/j.vaccine.2015.04.036

[13] Dube E Laberge C Guay M Bramadat P Roy R Bettinger J. Vaccine hesitancy: an overview. Hum Vaccin Immunother. (2013) 9:1763–73. 10.4161/hv.24657 23584253 PMC3906279

[14] Wu J Zhao L Wang M Gu J Wei W Li Q. Guardians' willingness to vaccinate their teenagers against COVID-19 in China: a national cross-sectional survey. J Affect Disord. (2022) 299:196–204. 10.1016/j.jad.2021.12.002 34875283 PMC8654519

[15] China NHcotPsRo. Knowledge on COVID-19 prevention and control 2021. Available online at: http://www.nhc.gov.cn/xcs/kpzs/list_gzbdfkzs.shtml

[16] China NHcotPsRo. COVID-19 Diagnosis and Treatment Protocol (Trial Eighth revised Edition) 2021. Available online at http://www.nhc.gov.cn/yzygj/s7653p/202104/7de0b3837c8b4606a0594aeb0105232b/files/f192ac6e5567469db4f0a8691ca18907.pdf

[17] Wang K Wong EL Ho KF Cheung AW Yau PS Dong D. Change of willingness to accept COVID-19 vaccine and reasons of vaccine hesitancy of working people at different waves of local epidemic in Hong Kong, China: repeated cross-sectional surveys. Vaccines. (2021) 9:62. 10.3390/vaccines9010062 33477725 PMC7832291

[18] Yang K Liu H Ma L Wang S Tian Y Zhang F. Knowledge, attitude and practice of residents in the prevention and control of COVID-19: an online questionnaire survey. J Adv Nurs. (2021) 77:1839–55. 10.1111/jan.14718 33259651 PMC7753640

[19] Chen Y Jin YL Zhu LJ Fang ZM Wu N Du MX. The network investigation on knowledge, attitude and practice about COVID-19 of the residents in Anhui Province. Zhonghua yu fang yi xue za zhi Chinese J Preventive Med. (2020) 54:367–73. 10.3760/cma.j.cn112150-20200205-00069 32268643

[20] Raina MacIntyre C Jay Hasanain S. Community universal face mask use during the COVID 19 pandemic-from households to travelers and public spaces. J Travel Med. (2020) 27:56. 10.1093/jtm/taaa056 32307526 PMC7188149

[21] Howard J Huang A Li Z Tufekci Z Zdimal V van der Westhuizen HM. An evidence review of face masks against COVID-19. Proc Natl Acad Sci USA. (2021) 118:4118. 10.1073/pnas.2014564118 33431650 PMC7848583

[22] Azlan AA Hamzah MR Sern TJ Ayub SH Mohamad E. Public knowledge, attitudes and practices towards COVID-19: a cross-sectional study in Malaysia. PLoS ONE. (2020) 15:e0233668. 10.1371/journal.pone.0233668 32437434 PMC7241824

[23] Fang Y Liu P Gao Q. Assessment of Knowledge, Attitude, and practice toward COVID-19 in China: an online cross-sectional survey. Am J Trop Med Hyg. (2021) 104:1461–71. 10.4269/ajtmh.20-0452 33606668 PMC8045651

[24] Alkhaldi G Aljuraiban GS Alhurishi S De Souza R Lamahewa K Lau R. Perceptions towards COVID-19 and adoption of preventive measures among the public in Saudi Arabia: a cross sectional study. BMC Public Health. (2021) 21:1251. 10.1186/s12889-021-11223-8 34187425 PMC8240080

[25] Wang J Jing R Lai X Zhang H Lyu Y Knoll MD. Acceptance of COVID-19 Vaccination during the COVID-19 Pandemic in China. Vaccines. (2020) 8:482. 10.3390/vaccines8030482 32867224 PMC7565574

[26] Wang J Lu X Lai X Lyu Y Zhang H Fenghuang Y. The changing acceptance of COVID-19 vaccination in different epidemic phases in China: a longitudinal study. Vaccines. (2021) 9:30191. 10.3390/vaccines9030191 33668923 PMC7996493

[27] Xiaoxiao Wang Leyuan Liu Minyue Pei Xiaoguang Li Nan Li. Willingness of the general public to receive a COVID-19 vaccine booster — China, April–May 2021. China CDC Weekly. (2022) 4:66–70. 10.46234/ccdcw2022.013 35186370 PMC8837444

[28] Abedin M Islam MA Rahman FN Reza HM Hossain MZ Hossain MA. Willingness to vaccinate against COVID-19 among Bangladeshi adults: understanding the strategies to optimize vaccination coverage. PLoS ONE. (2021) 16:e0250495. 10.1371/journal.pone.0250495 33905442 PMC8078802

[29] Wu J Li Q Silver Tarimo C Wang M Gu J Wei W. COVID-19 vaccine hesitancy among chinese population: a large-scale national study. Front Immunol. (2021) 12:781161. 10.3389/fimmu.2021.781161 34912346 PMC8666422

[30] Lin Y Hu Z Zhao Q Alias H Danaee M Wong LP. Understanding COVID-19 vaccine demand and hesitancy: a nationwide online survey in China. PLoS Negl Trop Dis. (2020) 14:e0008961. 10.1371/journal.pntd.0008961 33332359 PMC7775119

[31] Mohamad E Tham JS Ayub SH Hamzah MR Hashim H Azlan AA. Relationship between COVID-19 information sources and attitudes in battling the pandemic among the malaysian public: cross-sectional survey study. J Med Internet Res. (2020) 22:e23922. 10.2196/23922 33151897 PMC7674144

[32] Piltch-Loeb R Savoia E Goldberg B Hughes B Verhey T Kayyem J. Examining the effect of information channel on COVID-19 vaccine acceptance. PLoS ONE. (2021) 16:e0251095. 10.1371/journal.pone.0251095 33979370 PMC8116041

[33] Abdelhafiz AS Mohammed Z Ibrahim ME Ziady HH Alorabi M Ayyad M. Knowledge, perceptions, and attitude of Egyptians towards the novel coronavirus disease (COVID-19). J Community Health. (2020) 45:881–90. 10.1007/s10900-020-00827-7 32318986 PMC7173684

[34] Wang C Han B Zhao T Liu H Liu B Chen L. Vaccination willingness, vaccine hesitancy, and estimated coverage at the first round of COVID-19 vaccination in China: a national cross-sectional study. Vaccine. (2021) 39:2833–42. 10.1016/j.vaccine.2021.04.020 33896661 PMC8043613

[35] Wang Q Xiu S Zhao S Wang J Han Y Dong S. Vaccine hesitancy: COVID-19 and influenza vaccine willingness among parents in Wuxi, China-a cross-sectional study. Vaccines. (2021) 9:342. 10.3390/vaccines9040342 33916277 PMC8066309

